# A comparative evaluation of the efficacy of complete decongestive therapy in the treatment of unilateral breast cancer–related lymphedema with and without metabolic syndrome

**DOI:** 10.1007/s00520-024-08676-z

**Published:** 2024-06-29

**Authors:** Cansu Sahbaz Pirincci, Oguzhan Mete, Mustafa Ertugrul Yasa, Meltem Dalyan

**Affiliations:** 1 University of Health Sciences, Gulhane Faculty of Physiotherapy and Rehabilitation, Ankara, Türkiye; 2 Ankara City Hospital, Physical Medicine and Rehabilitation, Ankara, Türkiye

**Keywords:** Metabolic syndrome, Lymphedema, Complete decongestive therapy

## Abstract

**Aim:**

This study aimed to investigate the effect of the presence of metabolic syndrome (MetS) on the limb volume and quality of life (QoL) of patients who underwent complex decongestive therapy (CDT) due to unilateral breast cancer-related lymphedema (BCRL).

**Methods:**

Forty female patients with unilateral BCRL, of whom 20 had MetS (MetS group) and 20 did not have MetS (control group), were included in the study. The participants received CDT 5 days a week for 3 weeks. The participants’ limb volume (percentage of excess volume (PEV) and percentage reduction of excess volume (PREV) was determined using a tape measure, and their QoL was assessed using the Lymphedema Quality of Life questionnaire (LYMQoL) before and after treatment.

**Results:**

After the treatment, the PEV and PREV values and LYMQoL-symptoms scores of the patients improved (*p* < 0.05); however, the LYMQoL-function, appearance/body image, mood/emotions, and overall QoL scores did not change in the MetS group (*p* > 0.05). In the control group, the PEV and PREV values and the LYMQoL-appearance/body image, mood/emotions, and overall QoL scores improved (*p* < 0.05), but the LYMQoL-symptoms and LYMQoL-function scores did not change (*p* > 0.05). There was a greater increase in the post-treatment PEV and PREV values of the control group compared to the MetS group (*p* < 0.001).

**Conclusion:**

The study yielded that CDT was an effective treatment in BCRL with and without MetS; however, the improvement was greater in BCRL cases without MetS than in those with MetS. Therefore, the presence of MetS should be taken into account in the treatment of lymphedema in patients who develop BCRL.

**Trial registration:**

ClinicalTrials.gov, *identifier*: NCT05426993. Registered 2022–06-16. https://clinicaltrials.gov/search?cond=NCT05426993

## Introduction

Rather than representing a disease in and of itself, metabolic syndrome (MetS) is an umbrella term used for a set of risk factors associated with this condition. Alberti et al. proposed a global definition of MetS, which is still one of the most widely used criteria. According to this definition, an individual with three or more of the following five criteria is considered to have MetS: hypertension, dyslipidemia (hypertriglyceridemia and reduced levels of high-density lipoprotein cholesterol), hyperglycemia, and central obesity [1]. MetS has been recognized as one of the most significant risk factors for the twenty-first century epidemic of type 2 diabetes and cardiovascular disease [2, 3]. It is considered one of the most common chronic diseases and the fourth leading cause of death, with a global prevalence of 10.0 to 84.0% [4]. Lifestyle risk factors, such as obesity, smoking, inactivity, high consumption of refined carbohydrates, particularly elevated fructose, and saturated fat-rich diets, are potentially important causes of MetS [5, 6].

Lymphedema is a persistent, complex condition that develops when the lymphatic system’s capacity for transport falls below the normal lymphatic load, causing an accumulation of protein-rich interstitial fluid within extravascular spaces [7]. Swelling and a subsequent feeling of heaviness in the affected area can cause aesthetic impairment as well as reduced mobility and function. Lymphedema is typically divided into primary and secondary forms according to the etiology of the disease. Primary lymphedema is most commonly caused by developmental lymphatic anomalies, affecting patients of any age, whereas secondary lymphedema develops from injury or blockage of the lymphatics due to a disease or iatrogenic causes [7]. Primary lymphedema is far less prevalent in adults than secondary lymphedema, accounting for less than 1% of all events of lymphedema [8]. The most common lymphedema type is secondary lymphedema, which occurs in the upper extremities after breast cancer surgery [9]. Breast cancer–related lymphedema (BCRL) is the particular cause of secondary lymphedema in Western countries [10, 11]. Breast cancer is the most common cancer among Turkish women, similar to worldwide [12]. In addition, studies have reported that one in every five women develops lymphedema after breast cancer surgery. Patients with BCRL are the most frequent visitors to the clinic seeking treatment for lymphedema [13]. The key component of lymphedema treatment is complete decongestant therapy, also known as complex decongestive therapy (CDT), which comprises skin care, exercise, weight loss, compression, and manual lymphatic drainage (MLD). There are two phases of CDT. The first (decongestion) phase entails daily MLD and compression bandaging to achieve decongestion, a process that may take up to six weeks. The second (maintenance) phase aims to preserve and improve the outcomes of the first phase [14].

A study conducted among patients with BCRL suggested a connection between secondary lymphedema and MetS, primarily due to the notable association between insulin resistance and central obesity [15]. Furthermore, the study highlighted the significance of elevated serum leptin levels and disruptions in the adipocytokine signaling pathway in relation to lymphedema [16]. These findings help elucidate the link between altered lipid transport and the accumulation of fat in lymphedema. Additionally, Khairunnisa et al. demonstrated an association between secondary lymphedema in breast cancer survivors and features consistent with MetS, including obesity, hypertension, and diabetes mellitus. This association was supported by the analysis of circulating microRNA and adipokine levels [17]. Chakraborty et al. highlighted the presence of a link between lymphatic circulation and metabolic syndrome, stating that dysfunction in the lymphatic system could cause lipid transport and fat storage changes [18]. Besides, the efficacy of CDT is already known to be negatively impacted by obesity, which is a major indicator of MetS [19]. Another feature of metabolic syndrome is chronic low-grade inflammation. Pro-inflammatory cytokines related to metabolic syndrome may negatively affect vascular permeability. This increases the retention of lymphatic fluid [20]. Another factor that increases fluid retention is insulin resistance. If glycemic control cannot be achieved with insulin resistance, increased fluid retention may develop, which leads to tissue inflammation [20]. Hypertension, another factor of metabolic syndrome, can also worsen the lymphedema picture. With hypertension, increased pressure in the capillaries causes more fluid to pass between tissues and more fluid to accumulate in the interstitial space. In addition, in a study, hypertensive individuals were found to have lower vascular endothelial growth factor-C (VEGF-C) levels and it was emphasized that low VEGF-C levels cause impairment in lymph angiogenesis [21]. Breast cancer patients with MetS are at increased risk of developing lymphedema due to the combined effects of inflammation, lymphatic dysfunction, and metabolic disorders, and lymphedema is difficult to control [15]. However, to date, no research has been undertaken to directly compare the efficacy of CDT in the treatment of lymphedema with and without MetS. The current study was designed to investigate how the presence of MetS affected the outcomes of CDT in patients with BCRL. We hypothesized that patients with BCRL who had MetS would benefit less from CDT in terms of limb volume and quality of life (QoL) outcomes compared to those who did not have MetS.

## Methods

This study was conducted with 40 volunteer female patients with unilateral BCRL who presented to the Lymphedema Clinic of Ankara City Hospital between June 2022 and June 2023. Before the study, ethical approval was obtained from the Clinical Research Ethics Committee of the hospital (approval number: E2-22–1805). It was registered at http://clinicaltrials.gov (NCT05426993). All the participants were informed about the study, and their consent was obtained. The study was carried out by the tenets of the Declaration of Helsinki.

A total of 40 female patients aged 18 to 65 years who developed BCRL were included in the study. The participants were divided into two groups: those with MetS (MetS group, *n* = 20) and those without MetS (control group, *n* = 20). The diagnosis of MetS was made by the physician during the examination, taking into account the patient’s current and past examination findings. When making this diagnosis, the following National Cholesterol Education Program/Adult Treatment Panel III (NCEP-ATP III) consensus criteria were taken as a reference [22]: abdominal obesity (waist circumference ≥ 102 cm in men and ≥ 88 cm in women), the presence of hypertriglyceridemia (≥ 150 mg/dl) or receiving pharmacological treatment for high triglyceride levels, the presence of low high-density lipoprotein (HDL) (< 40 mg/dl in men and < 50 mg/dl in women) or receiving treatment for low HDL, hypertension (blood pressure ≥ 130/85 mmHg) or receiving anti-hypertensive treatment, and hyperglycemia (fasting blood glucose ≥ 100 mg/dl) or receiving treatment for high blood sugar levels.

The inclusion criteria for both groups were as follows: having unilateral BCRL, having stage 1–3 lymphedema according to the International Lymphedema Association criteria [14], being aged 18–65 years, having no cooperation problems, and agreeing to participate in the study. Additionally, for the MetS group, the presence of at least three of the above-mentioned NCEP-ATP III consensus criteria was sought. The exclusion criteria for both groups were refusing to participate in the study, having primary lymphedema, having active metastasis, receiving ongoing chemotherapy or radiotherapy, having mental cognitive problems, having bilateral BCRL, having previously received CDTs, and having an open skin wound.

### Evaluations

The participants’ demographic and clinical characteristics, including age, height, weight, body mass index (BMI), dominant extremity, lymphedema stage, type of surgery, lymphedema duration, number of chemotherapies or radiotherapies cures, and affected limb were recorded.

Limb volume and QoL evaluations of the individuals in both groups were undertaken by the same physician before treatment and 15 days after treatment. Edema was evaluated by measuring the affected and intact extremities with a tape measure at 4-cm intervals from the styloid protrusion of the ulna to the axilla. The volumes of the extremities were determined in cm^3^ based on the values obtained using the frustum formula. Absolute limb volume was calculated by subtracting the healthy limb volume from the affected limb. The severity of lymphedema was assessed based on the percentage of excess volume (PEV) calculated as follows: (affected limb volume − healthy limb volume)/healthy limb volume × 100%. PEV is better than the absolute difference in limb volume for describing lymphedema severity since this method minimizes the effects of BMI on the evaluation. The efficacy of CDT was measured as the percentage reduction of excess volume (PREV), formulated as 100% × [(initial − post-treatment limb volume)/excess volume. If PREV is 100%, this means that the volume of the arm with lymphedema has been successfully reduced to the level of the healthy arm [23].

QoL was evaluated using the Lymphedema Quality of Life (LYMQoL) questionnaire. The first 20 questions on this scale are scored from 1 to 4. This part has four subcategories: function, appearance, symptoms, and emotional state. The score for each subcategory is obtained by summing the score of each response and dividing the resulting score by the number of questions answered. A high score indicates poorer QoL. Question 21 evaluates the overall QoL and is scored between 0 and 10. A high score given to the last question indicates good QoL [24].

### Intervention

The patients underwent a total of 15 sessions of CDT (5 days a week, 3 weeks). This therapy consisted of MLD, skin care, compression bandages, and remedial exercises. Remedial exercises are a special exercise method applied in the treatment of lymphedema, planned to activate rhythmic, serial muscle groups. It includes active, rhythmic, repetitive, non-resistance exercises combined with breathing. These exercises consisted of diaphragmatic breathing exercise, range of motion, and isometric exercises for all extremities (shoulder, elbow, wrist, and fingers) [25]. MLD was applied to the patients as follows: Lymph drainage was applied first to the neck and then to the abdomen. After the stimulation of the central lymph nodes, the collateral axilla-axillary route was created by stimulating the axillary lymph nodes in the contralateral region, and the axilla-inguinal lymph route was created by stimulating the inguinal lymph nodes in the ipsilateral region. The patients were placed in a supine position, and the same routes were created from the posterior. Then, drainage was applied to the affected limb in the supine position. During this process, first, the proximal part was drained, gradually moving toward the distal part. The lymph fluid was drained from distal to proximal and delivered to the axillary and inguinal lymph nodes via the collateral pathways. The MLD application took approximately 30 min. Following MLD, skin care was performed to reduce skin tension, and then, a multilayer compression bandage was applied. This bandaging method was developed for lymphedema patients and involves the use of short-stretch bandages. The patients were asked not to remove the bandage from the extremity until they came to the clinic the following day for the next treatment session. Lastly, upper-extremity remedial exercises were performed to stimulate the lymphatic system. All CDT steps were undertaken by a physiotherapist (Foeldi College, Germany) with a lymphedema treatment certificate [26].

### Sample size

Five participants from each group were randomly recruited for the pilot study, and PREV was used to determine the required sample size for the study. The sample size was calculated using the G*Power software package (G^*^Power, Version 3.0.10, Franz Faul, Universität Kiel, Germany) [27]. The effect size (*d*) was calculated using pilot data with the G*Power software package. The effect size values of (*d*) =  < 0.2, 0.5, and 0.8 are interpreted as small, medium, and large effects, respectively [28]. According to this calculation, the effect size of *d* (effect size) was 1.159 (large effect size) for PREV. The results indicated that 19 participants were needed for each group (38 patients in total) to obtain 90% power and *α* = 0.05 type I error, considering a possible dropout rate of 10% [27].

### Statistical analysis

For analyses and calculations, a statistical analysis program (IBM SPSS Statistics for Windows, Version 22.0. IBM Corp., Armonk, New York, USA) was used. An overall *p*-value below 0.05 was considered statistically significant. The normality distribution of continuous variables was examined using histogram graphs, skewness and kurtosis coefficients, the Shapiro–Wilk test, coefficient of variance analysis, and detrended normal Q-Q graphs. Normally distributed variables were presented as mean and standard deviation, non-normally distributed variables as median and interquartile range, and ordinal variables as number and percentage. For the comparison of categorical variables, the chi-square test was used. For the inter-group comparison of continuous variables, the independent-sample *t*-test was used when the assumption of the bivariate normal distribution was met, and the Mann–Whitney *U* test was used otherwise. For the intra-group comparison of continuous variables, the paired-sample *t*-test was used in the case of a bivariate normal distribution, and the Wilcoxon singed-rank test was used in the case of a non-normal distribution. Using statistical software, the effect size (*d*) was calculated according to the *t* value and (*r*) for parametric tests and according to the *z* value for non-parametric tests [29]. The effect size values of (*d*) = 0.2, 0.5, and 0.8 and (*r*) = 0.1, 0.24, and 0.37 were interpreted as small, medium, and large effects, respectively [28].

## Results

**Fig. 1:**
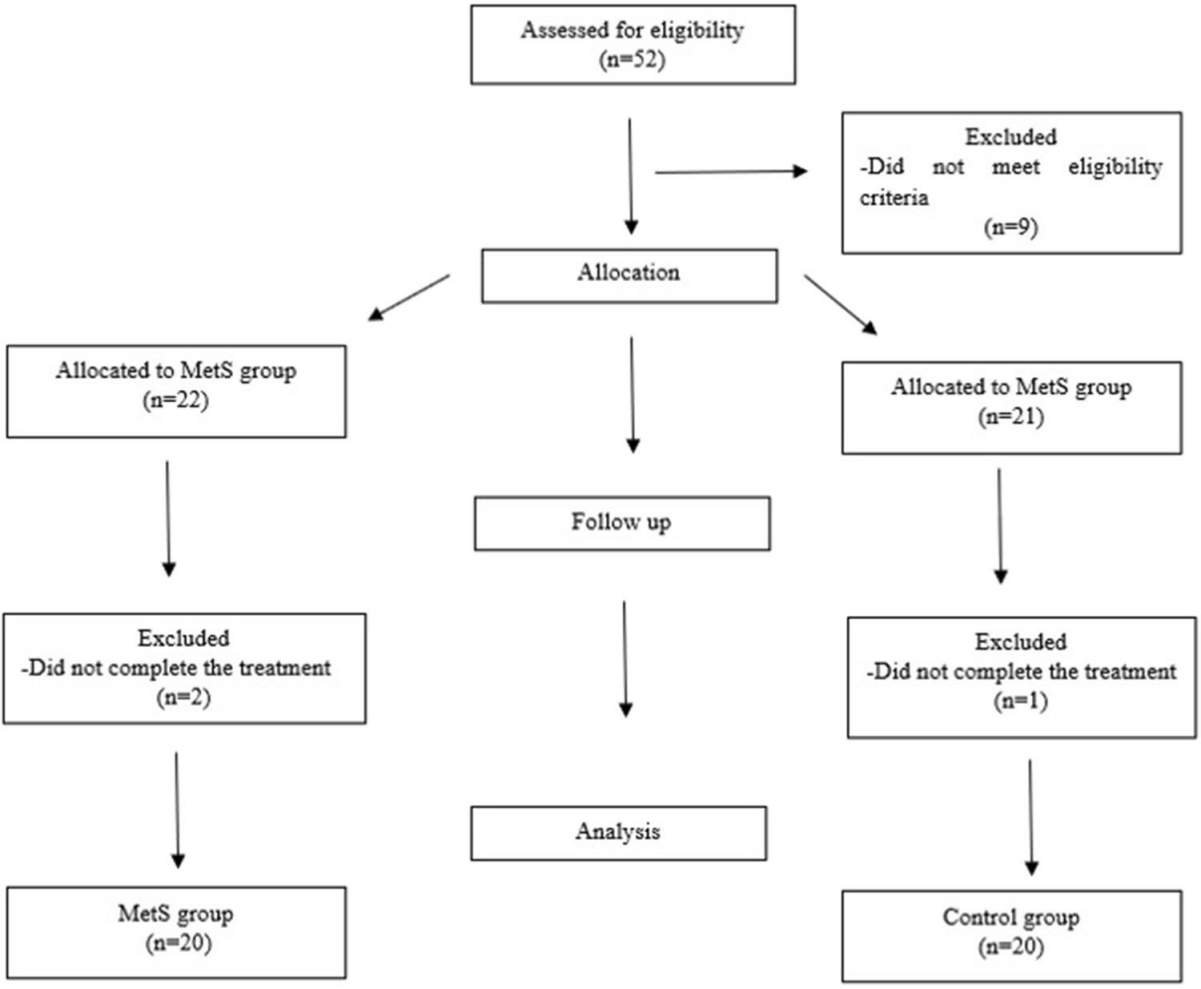
Flowchart of the participants

Fifty-two participants with BCRL were involved in the enrollment process, but five participants from the MetS group and four participants from the control group were not included in the sample because they did not meet the eligibility criteria. As a result, 43 patients with BCRL received CDT; however, two participants from the MetS group and one participant from the control group did not complete the treatment. Therefore, the study was finalized with 40 participants (20 from each group). The flowchart diagram of the participants is presented in Fig. 1. The participants did not report any adverse events related to the interventions performed during the treatment.

### Demographic and clinical characteristics

The two groups had similar demographic (age and BMI) and clinical (lymphedema duration, number of chemotherapies and radiotherapies cures, lymph dissection, operation date, and affected side) characteristics (*p* > 0.05) (Table 1).

**Table 1:** Comparison of the demographic and clinical characteristics of the groups

	MetS group (*n* = 20)	Control group (*n* = 20)	*p*
Age (year), *X* ± SD	53.60 ± 6.62	50.00 ± 9.38	0.170^a^
Body mass index (kg/m^2^), *X* ± SD	38.19 ± 8.09	30.42 ± 4.53	0.001^a^*
Lymphedema duration (month), median (IQR)	12.00 (24.00)	12.00 (48.00)	0.398^b^
Number of chemotherapies (cures), median (IQR)	7.00 (10.75)	6.00 (5.50)	0.547^b^
Number of radiotherapies (cures), median (IQR)	22.50 (5.00)	20.00 (9.50)	0.841^b^
Number of lymph dissection, median (IQR)	11.00 (7.25)	11.00 (10.75)	0.904^b^
Operation date (year)	4.00 (4.75)	4.00 (5.75)	0.404^b^
Affected side			
Right	11 (55%)	14 (85%)	0.327^c^
Left	9 (45%)	6 (15%)	
Lymphedema stage			
Stage 1	1 (5%)	6 (30%)	0.109^c^
Stage 2	14 (70%)	11 (55%)	
Stage 3	5 (25%)	3 (15%)	

*n* number (frequency), *X* mean, *SD* standard deviation, *IQR* interquartile range, *kg/m*^*2*^ kilogram/meter square

*p < 0.05

^a^Independent-samples *t*-test

^b^Mann-Whitney *U* test

^c^Chi-square test

### PEV and PREV

At the baseline, there were no differences between groups regarding PEV score (*p* = 0.904). The PEV score in the MetS group (*p* < 0.001; *r* 0.876) and the control group (*p* < 0.001; *r* 0.876) improved after treatment (Table 2). After treatment, there was a greater increase in the PEV value in the control group compared to the MetS group (*p* < 0.001; *r* 0.628) (Table 3).

**Table 2:** Intra-group comparison of PEV values and LYMQoL scores

	MetS group (*n* = 20)	Control group (*n* = 20)
PEV (median (IQR))		
Pre-treatment	16.48 (28.74)	14.05 (12.08)
Post-treatment	12.55 (27.37)	5.54 (2.55)
IG *p*-value	** < 0.001**^a^*	** < 0.001**^a^*
Effect size (r)	0.876	0.876
LYMQoL-function (*X* ± SD)		
Pre-treatment	2.32 ± 0.89	1.87 ± 0.55
Post-treatment	1.93 ± 0.75	1.68 ± 0.58
IG *p*-value	0.080^b^	0.131^b^
Effect size (*d*)	0.471	0.344
LYMQoL-appearance/body image (median (IQR))		
Pre-treatment	2.50 (1.65)	1.90 (3.00)
Post-treatment	2.20 (1.58)	1.40 (0.95)
IG *p*-value	0.106^a^	**0.002**^a^*
Effect size (*r*)	0.361	0.686
LYMQoL-symptoms (median (IQR))		
Pre-treatment	2.74 (1.91)	2.24 (1.13)
Post-treatment	2.33 (0.96)	1.73 (1.30)
IG *p*-value	**0.014**^a^*	0.298^a^
Effect size (*r*)	0.546	0.232
LYMQoL-mood/emotions (*X* ± SD)		
Pre-treatment	2.22 ± 0.80	2.00 (0.98)
Post-treatment	1.99 ± 0.72	1.45 (1.05)
IG *p*-value	0.186^b^	**0.016**^a^*
Effect size (*d*, *r*)	0.301	0.540
LYMQoL-overall QoL (median (IQR))		
Pre-treatment	6.00 (1.75)	6.40 ± 1.90
Post-treatment	6.00 (4.00)	7.40 ± 1.27
IG *p*-value	0.749^a^	**0.008**^b^*
Effect size (*r*, *d*)	0.071	0.585

*PEV* percentage of excess volume, *LYMQoL*: Lymphedema Quality of Life, *IQR* interquartile range, *n* number (frequency), *X* mean, *SD* standard deviation, *IG* intragroup comparison

*p < 0.05

^a^Wilcoxon signed-rank test

^b^Paired-samples *t*-test

**Table 3:** Inter-group comparison of pre-treatment to post-treatment changes in PEV and LYMQoL scores

	MetS group (*n* = 20)	Control group (*n* = 20)	*p* value	Effect size (*d*, *r*)
PEV (median (IQR))	− 4.13 (3.28)	− 9.94 (10.34)	< 0.001^a^*	0.628 (*r*)
LYMQoL-function (*X* ± SD)	− 0.39 ± 0.94	− 0.19 ± 0.55	0.430^b^	0.252 (*d*)
LYMQoL-appearance/body image (*X* ± SD)	− 0.24 ± 0.90	− 0.40 ± 0.49	0.509^b^	0.212 (*d*)
LYMQoL-symptoms (median (IQR))	− 0.20 (1.19)	− 0.80 (1.18)	0.242^a^	0.132 (*r*)
LYMQoL-mood/emotions (*X* ± SD)	− 0.23 ± 0.75	− 0.37 ± 0.58	0.494^a^	0.218 (*d*)
LYMQoL-overall QoL (median (IQR))	0.00 (1.00)	1.00 (1.00)	0.183^b^	0.220 (*r*)

*PEV* percentage of excess volume, *LYMQoL* Lymphedema Quality of Life, *IQR* interquartile range, *n* number (frequency), *X* mean, *SD* standard deviation

*p < 0.05

^a^Mann-Whitney *U* test

^b^Independent-samples *t*-test

**Fig. 2:**
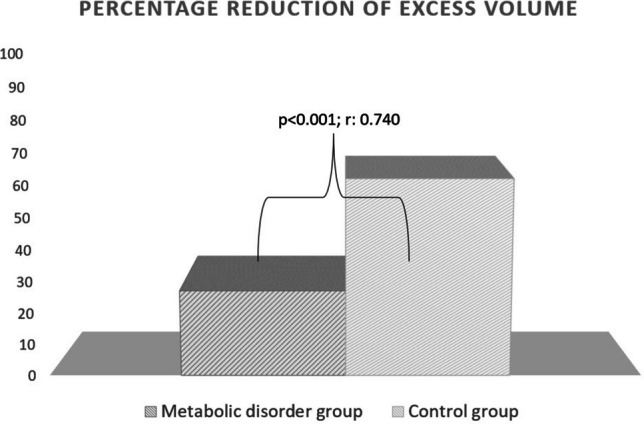
Comparison of percentage reduction of excess volume of the groups

At the end of the treatment, the change in the PREV value was 4.12 to 59.12% (median 28.82%) in the MetS group and 29.25 to 83.75% (median 61.50%) in the control group. The post-treatment change in the PREV value was significantly higher in the control group than in the MetS group (*p* < 0.001; *r* 0.740) (Fig. 2).

### Quality of life

At the baseline, there were no differences between groups in terms of LYMQoL-function (*p* = 0.067), LYMQoL-appearance/body image (*p* = 0.052), LYMQoL-symptoms score (*p* = 0.086), LYMQoL-mood/emotions (*p* = 0.631), except of LYMQoL-overall (*p* = 0.018). In the MetS group, the LYMQoL-symptoms score (*p* = 0.014; *r* 0.546) score improved after treatment; however, there were no significant changes in the LYMQoL-function (*p* = 0.080; *d* 0.471), LYMQoL-appearance/body image (*p* = 0.106; *r* 0.361), LYMQoL-mood/emotions (*p* = 0.186; *d* 0.301), or LYMQoL-overall QoL (*p* = 0.749; *r* 0.071) scores. In the control group, the LYMQoL-appearance/body image (*p* = 0.002; *r* 0.686), LYMQoL-mood/emotions (*p* = 0.016; *d* 0.540), and LYMQoL-overall QoL (*p* = 0.008; *d* 0.585) scores improved, while the LYMQoL-symptoms (*p* = 0.298; *r* 0.232) and LYMQoL-function (*p* = 0.131; *r* 0.344) scores did not significantly change (Table 2). After treatment, the changes in the LYMQoL-function (*p* = 0.430; *d* 252), LYMQoL-appearance/body image (*p* = 0.509; *d* 0.212), LYMQoL-symptoms (*p* = 0.242; *r* 0.132), LYMQoL-mood/emotions (*p* = 0.494; *d* 0.218), and LYMQoL-overall QoL (*p* = 0.183; *r* 0.220) scores were similar between the two groups (Table 3).

## Discussion

The current study showed that CDT was more effective in improving the PEV and PREV values in patients with unilateral BCRL who did not have MetS compared to those with MetS. In other words, limb volume changes in response to CDT were higher in the non-MetS group than in the MetS group. Moreover, CDT was observed to improve the PEV and PREV values and QoL in both groups.

The leading factor in the pathogenesis of MetS and its increasing prevalence is obesity [30]. Accumulated fat tissue can damage lymphatic system elements and negatively affect lymphatic drainage [31]. Due to obesity, lymphatic vessels may become compressed, and inflammation may occur in the lymphatic vessels. This may impede the transportation of lymph fluid from the proximal to the distal region, resulting in a disruption of lymph circulation [32, 33]. Obesity contributes to the development of hypertension, high serum cholesterol, decreased HDL cholesterol, and hypertriglyceridemia, which in turn contribute to the occurrence of other components of MetS [30]. The increased adipose tissue associated with obesity leads to an elevation in nonesterified fatty acids, cytokines, and plasminogen activator inhibitor-1, posing a risk to the body. Additionally, obesity is associated with a decrease in adiponectin levels, which play a role in clearing glucose, triglycerides, and free fatty acids from the bloodstream. In light of this information, obesity can be considered the most significant factor in the development of MetS [34]. A high fat tissue not only damages the lymphatic system but also negatively affects the efficacy of CDT. In a study, it was suggested that the impairment in lymphatic drainage of macromolecules in obese patients might cause a decrease in the response of CDT after axillary dissection [35]. On completion of the current study, we determined that although there was an improvement in limb volume after CDT in both groups, the recovery rate was greater in the group without MetS compared to the group with MetS. The greater efficacy of CDT in the non-MetS group can be attributed to the presence of the above-mentioned factors within this syndrome that hinder the efficacy of treatment, such as obesity and decreased lymph fluid transport.

Diabetes is another important component of MetS. It has been reported that diabetes causes an increase in lymphatic fluid, the glucose content of the fluid in the interstitial space is high, and the lymphatic fluid shows adhesive properties. In addition, it has also been reported that lymphatic function decompensates due to prolonged interstitial fluid overload due to hyperglycemia. These findings are evidence that the lymphatic system is affected in diabetic patients [20, 36]. Hypertension, another part of MetS, a risk bundle, also negatively affects lymphatics. A study has shown that serum VEGF-C levels are decreased in patients with hypertension, lymph angiogenesis, and the protective function of the lymphatic system are impaired. In addition, it has been suggested that decreased VEGF-C decreases the density of the capillary network [21, 37]. Low-grade inflammation in metabolic syndrome is another issue that should not be ignored. Low-grade inflammation causes systematic effects such as cell wall damage, proinflammatory increase, oxidative stress, loss of lipid control, and insulin resistance. All these inflammatory processes and their effects lead to dilation of lymphatic vessels, negative effects on vascular permeability, and incomplete functioning of lymphatics [20, 38]. As can be seen, MetS and its accompanying symptoms create a butterfly effect on the lymphatic system, causing it to be negatively affected. On completion of the current study, we determined that although there was an improvement in limb volume after CDT in both groups, the recovery rate was greater in the group without MetS compared to the group with MetS. The greater efficacy of CDT in the non-MetS group can be attributed to the presence of the above-mentioned factors within this syndrome that hinder the efficacy of treatment.

Effects of surgery and disease on QoL are evident in patients undergoing breast cancer surgery [39]. The accompanying presence of lymphedema in this scenario leads to a decline in the overall QoL of patients, bringing along further detrimental consequences associated with lymphedema [40]. Research suggests that patients who have undergone CDT have improved QoL. In a previous study, CDT was applied to 37 patients who developed BCRL, 5 days a week for 3 weeks, and there was an increase in patients’ QoL and functionality after treatment [41]. In another study, patients who developed BCRL received CDT 5 days a week for 4 weeks and were observed to have a reduction in limb volume and improvement in the overall QoL after treatment [42].

The findings of our study support the results reported in the literature. CDT improved some of the QoL parameters in both the MetS and non-MetS groups. There was no significant difference in QoL improvement between the two groups. Although CDT is effective in improving QoL, it can be stated that the presence of MetS does not have an additional effect on individuals treated for unilateral BCRL. We consider that the lack of a significant difference between the QoL of the patients with and without MetS may be related to the changes in QoL that may occur in the long term [43].

Our study has certain limitations. The long-term outcomes of CDT were not evaluated in patients who developed BCRL. Given that changes may occur in QoL in the long term, there is a need for future studies to evaluate the long-term efficacy of CDT, especially on QoL in this patient population. In addition, we only evaluated limb volume and QoL in the current study. It is also necessary to investigate changes in different parameters of CDT due to the presence of MetS among patients receiving CDT for BCRL. Finally, our study exclusively focused on patients with BCRL. Further research is warranted to investigate the impact of MetS on CDT outcomes in individuals with lymphedema in various anatomical regions, such as lower extremity lymphedema.

## Conclusion

The strength of this study is that it is the first to examine the effects of CDT on volumetric changes and QoL in patients with unilateral BCRL who did and did not have MetS. The results of the study showed that although CDT was an effective method in both patient groups, it was more effective on volumetric changes in those without MetS than in those with MetS. Thus, this study revealed that the presence of MetS affected the efficacy of CDT. We believe the clinical implications of this study is as follows. While CDT is widely acknowledged as the gold standard for lymphedema treatment, the effectiveness of this approach may be constrained by the presence of MetS. Hence, we advocate for the integration of MetS management strategies into lymphedema treatment protocols to optimize the efficacy of CDT for patients affected by BCRL.

## Data Availability

Not applicable.
